# Prevalence and associates of non-fatal overdose among people who inject drugs in Saveh, Iran

**DOI:** 10.1186/s13722-022-00325-2

**Published:** 2022-08-04

**Authors:** Bahram Armoon, Mark D. Griffiths, Azadeh Bayani, Rasool Mohammadi, Elaheh Ahounbar

**Affiliations:** 1 Social Determinants of Health Research Center, Saveh University of Medical Sciences, Saveh, Iran; 2 International Gaming Research Unit, Psychology Department, Nottingham Trent University, Nottingham, UK; 3 Student Research Committee, School of Allied Medical Sciences, Shahid Beheshti University of Medical Sciences, Tehran, Iran; 4 Department of Biostatistics and Epidemiology, School of Public Health and Nutrition, Lorestan University of Medical Sciences, Khorramabad, Iran; 5 Orygen, The National Center of Excellence in Youth Mental Health, University of Melbourne, Parkville, VIC Australia; 6 Center for Youth Mental Health, Faculty of Medicine, Dentistry and Health Sciences, University of Melbourne, Parkville, Australia; 7 School of Nursing and Midwifery, Saveh University of Medical Sciences, Shahid Beheshti Blvd, Shahid Fahmideh Blvd, 4th floor, Saveh, Markazi Province Iran

**Keywords:** Non-fatal overdose, People who inject drugs, Polysubstance use, Alcohol use, Psychological interventions

## Abstract

**Background:**

As a public health issue, non-fatal overdose (NFOD) is highly prevalent among people who inject drugs (PWID). This can lead to an elevated risk of future overdose, causing various harms including possible death. It is essential to improve knowledge concerning this problem and its associated risk factors to inform overdose prevention and assistance programs. The primary aim of the present study was to determine the prevalence of NFOD and associated risk factors among PWID in Saveh, Iran.

**Methods:**

In the present cross-sectional study, 272 PWID living in Saveh, Iran were interviewed face-to-face using a structured survey. Data concerning socio-demographics, substance use, risky behaviors, and services utilization data were collected. The outcome variable (i.e., NFOD) was assessed by answering “Yes” to the question: *“In the past three months, have you ever overdosed (at least once) by accident?”*

**Results:**

The prevalence of NFOD among PWID in the past three months was 54%. The characteristics and behaviors that were associated with an increased risk of experiencing NFOD in the past three months were being of older age (AOR = 5.2, *p* < 0.05), drug use initiation under the age of 22 years (AOR = 7.8, *p* < 0.05), being an alcohol user (AOR = 3.0, *p* < 0.05), and being a simultaneous multiple drug user (AOR = 5.8, *p* < 0.05). Also, more recent initiates to injecting (< 2 years) had an increased risk of experiencing a non-fatal overdose in the past three months. Findings also indicated that those who (i) attended a needle and syringe program (AOR: 0.3, *p* < 0.05), (ii) were visited by a general practitioner (AOR: 0.03, *p* < 0.05), and (iii) received a psychosocial intervention (AOR: 0.1, *p* < 0.05) were 0.3, 0.03 and 0.1 times less likely to report non-fatal overdosing than other participants, respectively.

**Conclusions:**

The results indicate that intervention and prevention initiatives seeking to reduce NFOD among PWID should not only be focused on the primary drug used but also the use of alcohol and polysubstance use. Specific and tailored psychological interventions combined with pharmacotherapy may be highly beneficial for PWID who experience more severe types of substance use, including alcohol use disorders and/or polysubstance abuse.

## Introduction

According to global statistics, there are 16 million people worldwide who inject drugs (PWID) [1]. A significant public health concern in Iran (where the present study was carried out) is drug injection [2]. Domestic data indicated that roughly 170,000-230,000 PWID live in Iran [3]. One study published in 2014 estimated that the prevalence of PWID in Iran was 0.43% [4]. Approximately one-third of all substance-induced deaths occur due to overdose among PWID using opioids [5].

It was reported that in 2017, there were 109,500 cases of opioid-induced overdose deaths, with the USA accounting for 43% of these [6]. Overall, a previous meta-analysis indicated that one-fifth of PWID have had an overdose in the preceding 12 months [7]. As a public health issue, non-fatal overdose (NFOD) is highly prevalent among PWID, and this can lead to an elevated risk of future overdose, causing various harms including possible death [8]. The frequency of NFOD is substantially greater than fatal overdose [9]. NFOD is associated with cardiac and renal conditions, traumatic injuries, and hypoxic brain injury [10, 11]. Warner-Smith et al. indicated that at least one of these complications is encountered in nearly 80% of individuals with an overdose experience [12]. There is also a significant association between enhanced healthcare costs and NFOD [13]. Injection drug use presents the highest biological vulnerability to overdose, compared to any other routes of consumption [7]. This is because PWID experience a more rapid substance action onset compared to individuals who apply other routes of substance administration, and for novice users, drug injection can be more risky and dangerous compared to more experienced users [14–16]. Injection drug use also contributes to a significant vulnerability to fatal and non-fatal overdose [17]. This is because PWID might deliberately or accidentally consume highly potent synthetic opioids, such as fentanyl [18, 19]. Fentanyl has similar pharmacologic effects as other mu opioid receptor agonists which include analgesia, euphoria, sedation, nausea and respiratory depression [20]. The increased use of synthetic opioids such as fentanyl and fentanyl analogs has changed the overdose epidemic in several ways. These synthetic opioids have increased the mortality risk for drug users, and the rate of overdose deaths doubled between 2015 and 2016, from 3.1 to 6.2 deaths per 100,000 [21]. Also, synthetic opioid use has increased deaths (i) in urban areas, (ii) among nonwhites, and (iii) among individuals in their 20 to 30 s [21].

Furthermore, characteristics, such as the administration of multiple substance types leading to increased risk of overdose (e.g., simultaneous use of alcohol and drugs; benzodiazepines and opioids), and polysubstance use significantly contribute to NFOD [3, 9, 17]. Neural depressants, such as alcohol or benzodiazepines, may have synergic interaction with opioids which can lead to overdose [22–26]. However, social context of drug use may affect the biological risks [27, 28]. When drug users undergo periods of forced abstinence during detoxification and who are released without medication therapy, the risk of overdose may be increased [29, 30]. Various factors, such as poverty, unstable residence, and current drug policies, may increase environmental risks and drug-related harms [31]. These situations may increase the risk of drug-related adverse effects, such as infectious disease and overdose [32–37].

To the best of the present authors’ knowledge, investigations on the prevalence and associated risk factors of NFOD among the Iranian population are limited [38, 39]. Moreover, no previous study has examined the associations between service use variables (such visits by a general practitioner [GP] and receiving a psychosocial intervention, and using needle and syringe programs [NSPs]), and non-fatal overdose among PWID. Better knowledge and use of these services are likely to reduce the likelihood of overdose among PWID [9, 40, 41]. Additionally, with NFOD increasing among PWID [7, 42], it is essential to improve knowledge regarding this problem and its associated risk factors to inform overdose prevention and assistance programs. Therefore, primary aim of this exploratory study was to determine the prevalence of NFOD and associated risk factors among PWID in Saveh, Iran.

## Methods

### Participant sample and context

The present study was conducted in Saveh, a city comprising approximately 220,000 individuals, in central Iran. NSP services are provided by two drop-in centers (DIC) in the city center of Saveh by outreach teams to PWID with poor access to DIC services. In the present cross-sectional study, 292 PWID were recruited. Of these, 150 were recruited from the drop-in services and the remainder were recruited utilizing snowball sampling. Ten participants were not eligible for consideration (because they did not have at least one illicit drug injection in the previous month), and another ten PWID did not want to participate in the study. Therefore, the final sample size was 272 (93% males and 7% females). Data were gathered via a structured interview utilizing a survey instrument which collected information concerning sociodemographic characteristics (e.g., gender, age, education) and substance use history (e.g., age of initiation, types of abused drugs during the past three months). The team of researchers kept in regular contact with PWID during the data collection period, and they asked the participants to encourage their peers to participate in the study by giving referral coupons (for which they could receive financial remuneration; details in the next section).

### Study site

The study setting was the harm reduction community-based DIC which is one of the two DICs which provide services to PWID under the supervision of Saveh University of Medical Sciences. All the study phases including recruitment, interview, HCV (Hepatitis C Virus) and HIV screening, diagnosis, and treatment were performed at this DIC. Community-based DICs in Saveh provide free opioid agonist treatment (OAT), sterile needles and syringes for injection, condoms, hot meals, and personal hygiene amenities. Additional services such as HIV screening and sexual health education are provided for those referred to DICs. Using respondent-driven sampling (RDS), 10 seeds (prominent and respected members of the PWID community introduced by the DIC team) were identified to recruit eligible individuals to the cross-sectional study between April 10, 2020, and May 1, 2021. Snowball sampling for recruiting additional participants was also employed. Injection drug use was confirmed by research staff through examining physical signs and markers of injection drug use, and the ability of participants to show knowledge of injection drug using behaviors. Participants who accepted to participate in the study received the equivalent of $3 US (∼150,000 Rials) as compensation for their time and to help with travel costs, and were given three coupons to encourage their peers to participate in the study. Participants then received an additional $2 US (∼100,000 Rials) for each participant they helped recruit. The study was a structured interview study using a survey to ask all the questions because some of the PWID did not have any education and would not have been able to complete the survey themselves. Also, PWID would have been unlikely to have completed such a survey in their own time from people that they do not know. Therefore, each participant was interviewed by staff at the drop-in center using the survey instrument.

### Procedure

At recruitment, eligible participants provided their informed consent and were then interviewed by same-gender interviewers. The data were collected by two staff members of the drop-in center and they personally knew all people who inject drug users in Saveh because they provided treatment for them. Therefore, we knew which participants were in which category and used each substance. In-person interviews with a survey instrument were performed in private rooms located at one of the DICs. Clinical psychologists working at the sites with a Master’s level qualification who had experience in working as addiction treatment providers and researchers conducted the interviews. All interviewers trained comprising a four-hour training session on performing in-depth interviews. Interviews ranged from 35 min to 2.5 h in length, averaging 72 min. Participants were given short breaks during interviews. GP visits, psychosocial consultation, laboratory assessments, and HCV treatment were supplied to them for free.

### Inclusion criteria

Participants who were over 18 years old and had at least one illicit drug injection in the previous month were considered to be eligible for the study. Injection status was confirmed by the existence track marks. Also, knowing the Farsi language was necessary to respond to the questions and to provide informed consent. Verbal or written informed consent was provided by all participants prior to the interview.

### Measures

Data were collected using face-to-face interviews. The study used the Bio-Behavioral Questionnaire (BBQ) in previous studies [3, 38]. The BBQ comprises four sections.

### Sociodemographic variables

This section of the BBQ assessed sex (male or female), age (less than 30 years old and more than 30 years old); educational attainment (less than high school education and high school graduates or higher education); employment status (yes or no); and income status (less than the equivalent of $50 US and more than $50 US per month).

### Substance use variables

This section of the BBQ assessed substance use in the last 30 days, including heroin, polysubstance, opioid, alcohol, cannabis use disorders, and methamphetamine use (binary: yes/no). All diagnoses identified were based on the Ninth Revision of the International Classification of Diseases (ICD-9) [43]. In the ICD-9, opioid use disorder and heroin use disorder are in the same category. However, in Iran, opioids (such as opium) are more available in Iran, so PWID are more likely to use opioids than heroin. Because our study was carried out under the supervision of the Iranian Ministry of Health of Iran, they needed more clarification and classification for health management and policy. Therefore, opioid use disorder and heroin use disorder were classified separately.

### Risky behaviors

This section of the BBQ assessed two risky behaviors, namely, the age of first injection of drugs (≤ 22 years and more than 22 years) and duration of drug injection behavior (less than two years and more than two years). The 22 years cut-point for early drug use was chosen based on previous studies that had done similarly [3, 44, 45].

### Service use variables

This section of the BBQ assessed three service use variables, namely, whether the participants had (i) used NSP services (which provided methadone maintenance therapy, a package containing four syringes/needles, four extra needles, water vials, filters, alcohol pads, and two condoms at each visit [46]), (ii) received visits from a GP (who checked on their general health including their drug use and HIV/HCV/HBV status, etc.), and (iii) received psychosocial interventions in the past three months (e.g., food, shelter and psychological interventions including motivational interviewing, a guided and client-based counselling process supported by the principles of cognitive and behavioral theory through which the therapist motivates the client to identify issues, concerns, emotions, beliefs and behaviors in each month during a three-month period). These were answered either ‘yes’ or ‘no’.

### Content and face validity of the interview questionnaire

Both content validity and face validity of the BBQ were assessed under the supervision of eight subject specialists (including epidemiologists and substance use experts). In addition, face validity was assessed using 10 PWID from the same region who did not participate into the main study. Internal consistency reliability for each scale was estimated: substance use variables (ɑ = 0.89), risky behaviors (ɑ = 0.82) and service use variables (ɑ = 0.85). The reliability was assessed by interviewing ten eligible people twice with a two-week period.

### Study outcome variable

The outcome variable (i.e., NFOD) was assessed by answering “Yes” to the question: *“In the past three months, have you ever overdosed (at least once) by accident?”* NFOD was defined using one or more of the following characteristics: loss of consciousness, blue skin color, collapsing, inability to wake up, encountering convulsions, experiencing difficulties with breathing, and myocardial infarction occurring during drug use. This definition was in accordance with previous studies conducted in Australia [47], USA [48], and Iran [38]. In the participants reported any of these characteristics, they were considered as PWID who experienced an overdose. The participants were asked to report any overdose experience over the past three months.

### Data analysis

To analyze the demographic variables, drug use histories, and NFOD histories of the participants, descriptive statistics were applied. First, the bivariate associations between all independent variables and the prevalence of NFOD using Pearson’s chi-square test were calculated. Both bivariate and multivariate logistic regression models were used to determine factors associated with NFOD among PWID. To identify factors associated with NFOD, variables with a *p*-value < 0.2 in the bivariate analyses were entered into multivariate logistic regression model. Collinearity statistics were tested using variance inflation factors (VIF) and tolerance tests. The results as adjusted odds ratio (AOR) and 95% confidence interval (CI) were reported. A *p*-value of 0.05 was considered statistically significant, and STATA v. 14 software was used for analysis.

## Results

The sample comprised 272 PWID, aged between 23 and 70 years (mean = 41.5 years, SD = 7.41). The majority of participants were more than 30 years old (83%), and had high school level of education (62%). Moreover, 81% earned more than $50 income per month, approximately 60% had heroin use and polysubstance use disorders, two-thirds of PWID were methamphetamine use disorders (65%), 71% had an opioid use disorder, and over half had an alcohol use disorder (55%). Over two-thirds of PWID reported their initiation into injecting drugs before the age of 22 years (70%), and two-thirds had less than two years of injecting drugs (64%). Additionally, 37% had attended NSPs, approximately half had been visited by their GP (43%), and 71% had received a psychosocial intervention. The prevalence of NFOD in the past three months was 54%. (Table 1). Among those who had an NFOD, the majority reported using heroin (60%), poly-substances (i.e., simultaneous use of two or more psychoactive substances) (71%), opioids (59%), cannabis (71%), alcohol (67%), and methamphetamine (66%). Two-thirds of those with an NFOD reported their initiation into injecting drug use before 22 years of age (68%) had less than two years of injection drug use, and approximately half had received a psychosocial intervention (49%).

**Table 1:** Characteristics of individuals who inject drugs and non-fatal overdose experience, Saveh, Iran, 2021

Characteristics	Non-fatal overdose in the past three months		*p*-value
	Yes (n = 149) N (%)	No (n = 123) N (%)	
Age			
≤ 30 years	14 (9)	31 (25)	0.001
> 30 years	135 (91)	92 (75)	
Sex			
Male	138 (93)	115 (93)	0.77
Female	11 (7)	8 (7)	
Education			
≤ High school	41 (27)	60 (49)	0.00
> High school	108 (73)	63 (51)	
Employment status			
Unemployed	68 (41)	63 (51)	0.39
Employed	81 (59)	60 (49)	
Income (US$)			
≤ 50$ per month	27 (18)	24 (19)	0.87
> 50$ per month	122 (82)	99 (81)	
Heroin use disorders			
Yes	96 (64)	62 (51)	0.02
No	53 (36)	61 (49)	
Polysubstance use disorders			
Yes	111 (75)	45 (36)	0.001
No	38 (25)	78 (64)	
Methamphetamine use disorders			
Yes	117 (78)	61 (49)	0.001
No	32 (22)	62 (51)	
Opioid use disorders			
Yes	116 (77)	79 (64)	0.01
No	33 (23)	44 (36)	
Cannabis use disorders			
Yes	97 (65)	39 (32)	0.001
No	52 (35	84 (68)	
Alcohol use disorders			
Yes	102 (68)	50 (41)	0.001
No	47 (32)	73 (59)	
Age of onset to injection (years)			
≤ 22	131 (88)	62 (51)	0.001
> 22	18 (12)	61 (49)	
Duration of inject drug (years)			
≤ 2	105 (70)	70 (57)	0.02
> 2	44 (30)	53 (43)	
Methadone treatment			
Yes	122 (9)	103 (25)	0.68
No	27 (91)	20 (75)	
Needle and syringe program use			
Yes	45 (30)	57 (46)	0.006
No	104 (70)	66 (54)	
General practitioner visits			
Yes	34 (22)	82 (67)	0.001
No	115 (78)	41 (33)	
Receiving psychosocial intervention			
Yes	95 (64)	98 (80)	0.004
No	54 (36)	25 (20)	

In the bivariate analyses, socio-demographics, drug use, risky behaviors, and service use were significantly associated with the past three months’ history of NFOD (Table 2). Being older than 30 years, high education level, heroin use, polysubstance use, opioid use, alcohol use, cannabis use disorder, and methamphetamine use were significantly associated with NFOD (*p* < 0.05). Initiation into injecting drug use before the age of 22 years and having less than two years of injection drug use were significantly associated with increased odds of NFOD (*p* < 0.05). However, attending NSPs, receiving GP visits, and receiving a psychosocial intervention were all associated with decreased odds of NFOD (*p* < 0.05) (Table 2).

**Table 2:** Bivariate and multiple logistic regression of factors associated with non-fatal overdose among PWID (last 3 months)

Characteristics	Bivariate COR (95% CI)	*p*-value	Multivariate AOR (95% CI)	*p-*value
Age				
≤ 30 years	1 (reference)	0.001	1 (reference)	0.004
> 30 years	3.24 (1.63–6.44)		5.24 (1.69–16.21)	
Education				
≤ High school	1 (reference)	0.000	1 (reference)	0.05
> High school	0.39 (0.24–0.66)		0.44 (0.18–1.03)	
Employment status				
Unemployed	0.80 (0.49–1.29)	0.35		
Employed	1 (reference)			
Income (US$)				
≤ 50$ per month	1 (reference)	0.77		
> 50$ per month	1.09 (0.59–2.01)			
Heroin use disorders				
Yes	1.78 (1.09–2.9)	0.02	1.43 (0.39–5.24)	0.58
No	1 (reference)		1 (reference)	
Polysubstance use disorders				
Yes	5.06 (3.01–8.51)	0.000	5.87 (2.47–13.93)	0.001
No	1 (reference)		1 (reference)	
Methamphetamine use disorders				
Yes	3.71 (2.19–6.29)	0.000	1.34 (0.5–3.56)	0.55
No	1 (reference)		1 (reference)	
Opioid use disorders				
Yes	1.95 (1.14–3.34)	0.01	1.44 (0.58–3.56)	0.42
No	1 (reference)		1 (reference)	
Cannabis use disorders				
Yes	4.01 (2.41–6.67)	0.000	2.03 (0.68–6.06)	0.2
No	1 (reference)		1 (reference)	
Alcohol use disorders				
Yes	3.16 (1.92–5.21)	0.000	3.01 (1.28–7.08)	0.01
No	1 (reference)		1 (reference)	
Age of onset to injection				
≤ 22	7.16 (3.9-13.13)	0.000	7.83 (3.01–20.34)	0.001
> 22	1 (reference)		1 (reference)	
Duration of inject drug (years)				
≤ 2	1.8 (1.09–2.98)	0.02	1.33 (0.54–3.27)	0.52
> 2	1 (reference)		1 (reference)	
Needle and syringe program use				
Yes	0.5 (0.3–0.82)	0.007	0.32 (0.14–0.75)	0.009
No	1 (reference)		1 (reference)	
General practitioner visits				
Yes	0.14 (0.08–0.25)	0.000	0.03 (0.009-0.1)	0.001
No	1 (reference)		1 (reference)	
Receiving psychosocial intervention				
Yes	0.44 (0.25–0.77)	0.004	0.17 (0.04–0.61)	0.007
No	1 (reference)		1 (reference)	

* COR* crude odds ratio, *AOR* adjusted odds ratio

In the final multiple logistic regression model **(**Table 2**)**, the characteristics and behaviors that were associated with an increased risk of experiencing an NFOD in the past three months are presented. There were no significant associations between NFOD and socioeconomic characteristics (education and income), some types of drug use (e.g., heroin use, opioid use, cannabis use disorder, methamphetamine use), and risky behavior (e.g., duration of injecting drugs). Among the sociodemographic variables, being older than 30 years (adjusted odds ratio [AOR]: 5.2, 95% [confidence interval] CI: 1.6–16.2) was significantly and independently associated with higher odds of an NFOD. The results also indicated that polysubstance use (AOR: 5.8, 95% CI: 2.4–13.9) and alcohol use disorders (AOR: 3, 95% CI: 1.2–7) were positively associated with NFOD among PWID. Results also showed that first injecting drugs under the age of 22 years was significantly associated with NFOD (AOR: 7.8, 95% CI: 3–20.3). Finally, the results showed that attending NSPs (AOR: 0.3, 95% CI: 0.1–0.7), visiting by GP (aOR: 0.03, 95% CI: 0.009-0.1), and receiving psychosocial interventions (AOR: 0.1, 95% CI: 0.04–0.6) were negatively associated with NFOD. Moreover, those attending NSPs, having visits by a GP, and receiving psychosocial interventions were 0.3, 0.03, and 0.1 times less likely to report non-fatal overdosing than other participants, respectively.

## Discussion

The present study assessed the sociodemographic characteristics, drug type, behavioral risk factors, and service use variables associated with non-fatal overdose (NFOD) among people who inject drugs (PWID) in Saveh (Iran). After adjustment, and in line with prior research findings, the findings indicated a significant relationship between an elevated NFOD risk and older age. A large body of literature has suggested that NFOD occurs among novice, inexperienced, or young substance users (as opposed to those who are middle-aged and older-aged). In other words, an overdose experience is typically experienced by individuals in their late twenties and early thirties including some who have considerable substance use experience in general [49, 50].

The findings indicated that an experience of NFOD in the previous three months was stronger among PWID with the illicit drug injection onset age of below 22 years. Substance dependence and multiple biopsychosocial issues are more frequent among individuals who initiate substance use at an early age. Therefore, it is essential to evaluate further the onset age of substance use [38, 51]. The inception age of opioid use is a major risk factor, and earlier research has suggested a significant association between younger onset of substance use and higher likelihood of substance dependence, causing further health and social-related problems [15, 45, 52–54].

Consistent with the previous empirical investigations, the results showed a positive association between NFOD and alcohol use disorders among PWID [50, 55]. Other studies have also argued that the frequency of drug overdose is higher among PWID reporting alcohol use disorder. Therefore, drinking alcohol appears to interact at behavioral and pharmacological levels [56]. Therefore, more attention must be paid to the substantial impact of alcohol drinking on overdose among PWID. Alcohol overdose is one of the major reasons for emergency department visits [57]. Although alcohol is not the only toxin in fatal overdoses [58], heavy alcohol use has an important negative effect on long-term morbidity and mortality [59]. Additionally, the impact of the use of central nervous system depressants such as alcohol on fatal and non-fatal overdose has been explained with PWID who inject opioids [13, 60, 61]. Respiratory depression is considered as a major cause of fatal heroin overdoses. Alcohol use is a relatively weak respiratory depressant [62]. However, heroin may increase the latter substance’s effect when is combined with the potent respiratory depressant [63]. Even small amounts of alcohol in combination with heroin may be obviously a risk factor of fatal consequences [64]. Alcohol use combined to a ‘safe’ dose of opioids may be fatal [65]. Consequently, it is crucial to incorporate awareness concerning abuse of alcohol and other substances in preventive programs and harm reduction settings targeting PWID in Iran.

The rates of NFOD were greater among PWID engaging in polysubstance use in the preceding one month. In this respect, a significant body of literature has demonstrated a robust association between polysubstance use and both fatal overdoses and NFODs [23, 48]. Merging central nervous system depressants or stimulants with opioids have each been related with higher risks of encountering a fatal overdose [66, 67]. Also, polysubstance users may experience more complex issues, including homelessness and heightened states of being violent [68].

The findings in the present study suggested a decreased risk of NFOD among PWID attending NSPs and concurs with the results of previous research [69]. This indicates that overdose can be prevented by integrating needle and syringe exchange programs with the provision of overdose education plus naloxone [70]. Additionally, other resources and healthcare plans consist of counseling services, substance use treatment, and Hepatitis C Virus (HCV)/HIV testing for PWID [71–74], along with providing naloxone combined with overdose training for PWID [75]. Also, integrating the healthcare system can significantly promote the conditions of PWID, leading to an improved society [76, 77]. NSPs are secondary prevention programs that can lead to reduced risk of injection drug use and its consequent burden. Furthermore, NSPs are associated with overdose prevention programs in society, safe disposal of used injection equipment, and selling non-prescription syringes in pharmacies [9, 78, 79].

A novel finding of the present study was that PWID who were visited by a GP or attended psychosocial interventions had a lower risk for experiencing an NFOD. On the other hand, service utilization had a relationship between multiple referrals to healthcare providers and GP visits and psychosocial intervention, which might be because patients felt supported and cared for following visits, i.e., associated with appropriate referrals, advice, and recovery suggestions for individuals who use psychoactive substances [80].

## Limitations

There are some limitations to the present study. The cross-sectional nature of the study prevented the establishment of any causal inferences between risk factors and drug overdose. Furthermore, the data were all self-report, which is subject to misclassification, recall bias, and/or social desirability bias. Although a specific definition of overdose was provided to the participants, their perception of overdose might have been different leading to a lack of consensus among PWUD in this regard among them. Moreover, the definition of NFOD used was arguably problematic. For most of what is included in this composite outcome, there would be no way for the participant to know if these events occurred unless they were injecting with a partner and were told about the events. Other indicators of NFOD might be (i) receiving an opioid antagonist, (ii having someone call for medical help, and (iii) feeling more sedated, drugged, or high than the individual wanted to be or felt was safe. As the study sample was not selected randomly, the collected data might not be generalizable to other populations of PWID. Moreover, the individuals in the present study might have underreported their overdose experiences because of being under the influence of potent substances, leading to failure to precisely recall the overdose events. Another limitation is that the study did not ask participants how often they accessed the NSP services (only if they had accessed them) and the frequency of accessing NSP services may have influenced the findings. Additionally, the use of snowball sampling also limits the ability to determine whether the prevalence of NFOD among PWID in Iran is accurate or if it is skewed some way. There may be PWID who do not associate with others or who did not use the services by which people were recruited. Moreover, because the present study only evaluated NFOD, it did not evaluate fatal overdose among this vulnerable population.

## Conclusions

The most positive significant associations with NFOD among PWID were being older than 30 years, age of drug injection onset under 22 years, polysubstance use, and alcohol use disorder. In contrast, the most negative associations with NFOD among PWID were attending NSPs, having visits from a GP, and receiving psychosocial interventions. The results suggest that intervention and prevention initiatives seeking to reduce NFOD among PWID should not only focus on the primary drug used but also the use of alcohol and polysubstance use. Specific and tailored psychological interventions combined with pharmacotherapy can be highly beneficial for PWID who experience more severe types of substance use, such as alcohol use disorders or polysubstance [81].

## Data Availability

The datasets used and/or analyzed during the present study are available from the corresponding author on reasonable request.

## Abbreviations

PWID: People who inject drugs
NFOD: Non-fatal overdose
GP: General practitioner
NSP: Needle and syringe program
DIC: Drop-in centers
OAT: Opioid agonist treatment
AOR: Adjusted odds ratio
CI: Confidence interval
HCV: Hepatitis C Virus
